# *IRF4* expression is low in Philadelphia negative myeloproliferative neoplasms and is associated with a worse prognosis

**DOI:** 10.1186/s40164-021-00253-y

**Published:** 2021-12-24

**Authors:** Cosimo Cumbo, Francesco Tarantini, Luisa Anelli, Antonella Zagaria, Immacolata Redavid, Crescenzio Francesco Minervini, Nicoletta Coccaro, Giuseppina Tota, Alessandra Ricco, Elisa Parciante, Maria Rosa Conserva, Giorgina Specchia, Pellegrino Musto, Francesco Albano

**Affiliations:** 1 Department of Emergency and Organ Transplantation (D.E.T.O.), Hematology and Stem Cell Transplantation Unit, University of Bari “Aldo Moro”, P.Zza G. Cesare, 11, 70124 Bari, Italy; 2 School of Medicine, University of Bari “Aldo Moro”, 70124 Bari, Italy

**Keywords:** *IRF4* expression, Philadelphia negative MPNs, Prognosis

## Abstract

Interferon regulatory factor 4 (*IRF4*) is involved in the pathogenesis of various hematologic malignancies. Its expression has been related to the negative regulation of myeloid-derived suppressor cells (MDSCs) and the polarization of anti-inflammatory M2 macrophages, thereby altering immunosurveillance and inflammatory mechanisms. An abnormal inflammatory status in the bone marrow microenvironment of myeloproliferative neoplasms (MPNs) has recently been demonstrated; moreover, in chronic myeloid leukemia a downregulated expression of *IRF4* has been found. In this context, we evaluated the *IRF4* expression in 119 newly diagnosed consecutive Philadelphia negative MPNs (Ph- MPNs), showing a low expression among the MPNs phenotypes with a more significant decrease in primary myelofibrosis patients. Lower *IRF4* levels were associated with *JAK2* + and triple negatives cases carrying the worst prognosis. Furthermore, the *IRF4* levels were related to leukemic transformation and a shorter leukemia-free survival; moreover, the risk of myelofibrosis transformation in polycythemia vera and essential thrombocythemia patients was more frequent in cases with lower *IRF4* levels. Overall, our study demonstrates an *IRF4* dysregulated expression in MPNs patients and its association with a worse prognosis. Further studies could validate these data, to improve our knowledge of the MPNs pathogenesis and confirm the *IRF4* role as a new prognostic factor.

**Supplementary Information:**

The online version contains supplementary material available at 10.1186/s40164-021-00253-y.

**To the Editor**

Interferon regulatory factor 4 (*IRF4*) is a transcription factor with an established role in the pathogenesis of various hematologic malignancies [1]. *IRF4* expression has been related to the negative regulation of myeloid-derived suppressor cells (MDSCs), thereby altering immunosurveillance; moreover, its expression drives inflammation through the polarization of anti-inflammatory M2 macrophages [2, 3]. In chronic myeloid leukemia a downregulated expression of *IRF4* has been found [4] and recent evidence supports its prognostic role in *JAK2*V617F mutated myeloproliferative neoplasms (MPNs) [5].

With these premises, we evaluated *IRF4* expression in 119 Philadelphia negative MPNs (Ph- MPNs) patients (Additional file 2: Table S1) to verify its role on clinical outcome (median follow-up: 61.5 months, range: 1–238). The quantification was calculated as the ratio between *IRF4* and *GUSB* number of copies (I/G) (Additional file 1).

The bone marrow (BM) *IRF4* median value was 0.11 I/G (min. 0.11- max. 012) and 0.04 I/G (min. 0.001 – max. 0.27) in the healthy and in the MPNs groups, respectively (p < 0.0001). Considering the *IRF4* median value for every MPN type, the difference compared to the healthy controls (HC) remained statistically significant (Fig. 1A). In particular, the primary myelofibrosis (PMF) patients showed a lower *IRF4* median value than that of the other groups compared to the HC. Among the MPNs, the PMF *IRF4* median value was lower than in the essential thrombocythemia (ET) group (p = 0.003). *CALR* mutated cases showed a higher *IRF4* expression than those with *JAK2* (0.06 vs 0.03, p = 0.007) or triple-negative (TN) (0.06 I/G vs 0.02 I/G, p = 0.0008) (Fig. 1B). *IRF4* expression was not associated with variables as sex, age, risk group [6]. Fourteen (11.7%) patients showed leukemic transformation (LT): 8 PMF, 4 secondary myelofibrosis (SMF), and 2 polycythemia vera (PV); they had a lower *IRF4* expression at diagnosis compared to the other MPN patients (0.01 I/G vs 0.04 I/G, p = 0.0005) (Fig. 1C). An optimal cutoff of the *IRF4* expression value best identifying the possibility of MPN leukemic transformation was defined by ROC analysis. The area under the curve was 0.79 (95% CI 0.71–0.86; p < 0.0001). Representative cutoff values for sensitivity and specificity were calculated and plotted on the curve. An optimal value of 0.022 I/G was obtained, with a sensitivity of 76.9% (95% CI 46.2–95.0) and a specificity of 76.1% (95% CI 66.9–84.0). This value distinguished MPN patients with a higher probability of LT; in fact, the group with an *IRF4* value < 0.022 I/G had shorter leukemia-free survival (LFS) (Fig. 2A). The LFS analysis was also considered only for the myelofibrosis (MF) group; patients with an *IRF4* value < 0.022 I/G showed a shorter LFS (p = 0.001, Fig. 2B). In another LFS analysis, this difference was confirmed when considering only the PMF group: the median LFS for PMF patients with *IRF4* < 0.022 I/G was 23 months, whereas LFS was not reached for the > 0.022 I/G group (p = 0.0009) (Fig. 2C). Overall survival (OS) analysis in the MF group showed that patients with *IRF4* > 0.002 I/G had longer median survival than those in the < 0.022 I/G group (143.9 mo. versus 40.5 mo., p = 0.04) (Fig. 2D). Also, in the PMF group, the *IRF4* value > 0.022 I/G was associated with a longer OS (75 mo versus 21 mo, p = 0.04) (Fig. 2E). Moreover, PV and ET patients with an *IRF4* value < 0.022 I/G showed a shorter time to MF transformation (109 mo. versus 190 mo., p = 0.03) (Fig. 2F).

**Fig. 1:**
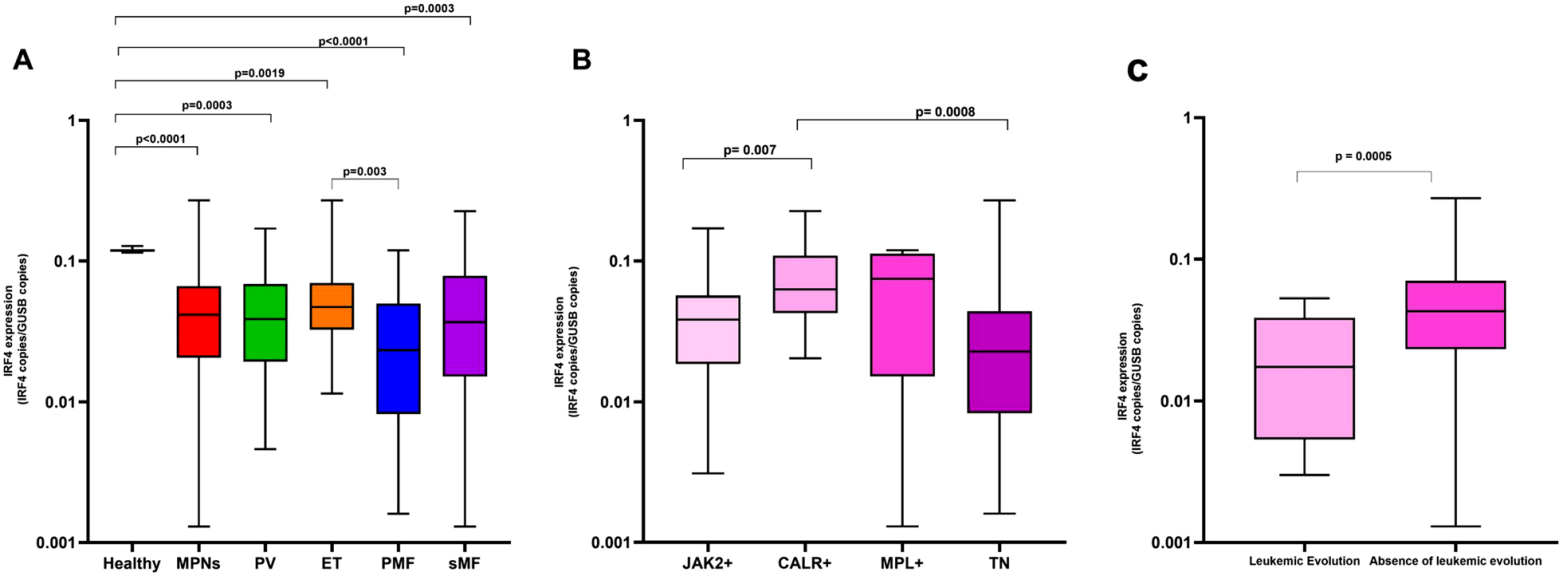
*IRF4* expression in Ph-MPNs. **A** Comparison of *IRF4* expression between healthy controls and MPN groups. The *IRF4* expression was statistically significantly lower in every MPNs category than in healthy controls. **B** *IRF4* expression according to the gene driver mutation in MPNs. A lower *IRF4* expression was more frequently concomitant with *JAK2* + and triple-negative status than with the *CALR* gene mutation MPN group. **C** The MPN patients group with leukemic transformation showed a lower *IRF4* expression than the group without leukemic evolution. Boxplots representing the distribution. The box always extends from the 25th to 75th percentiles. The line in the box represents the median. The whiskers go down to the smallest value and up to the largest

**Fig. 2:**
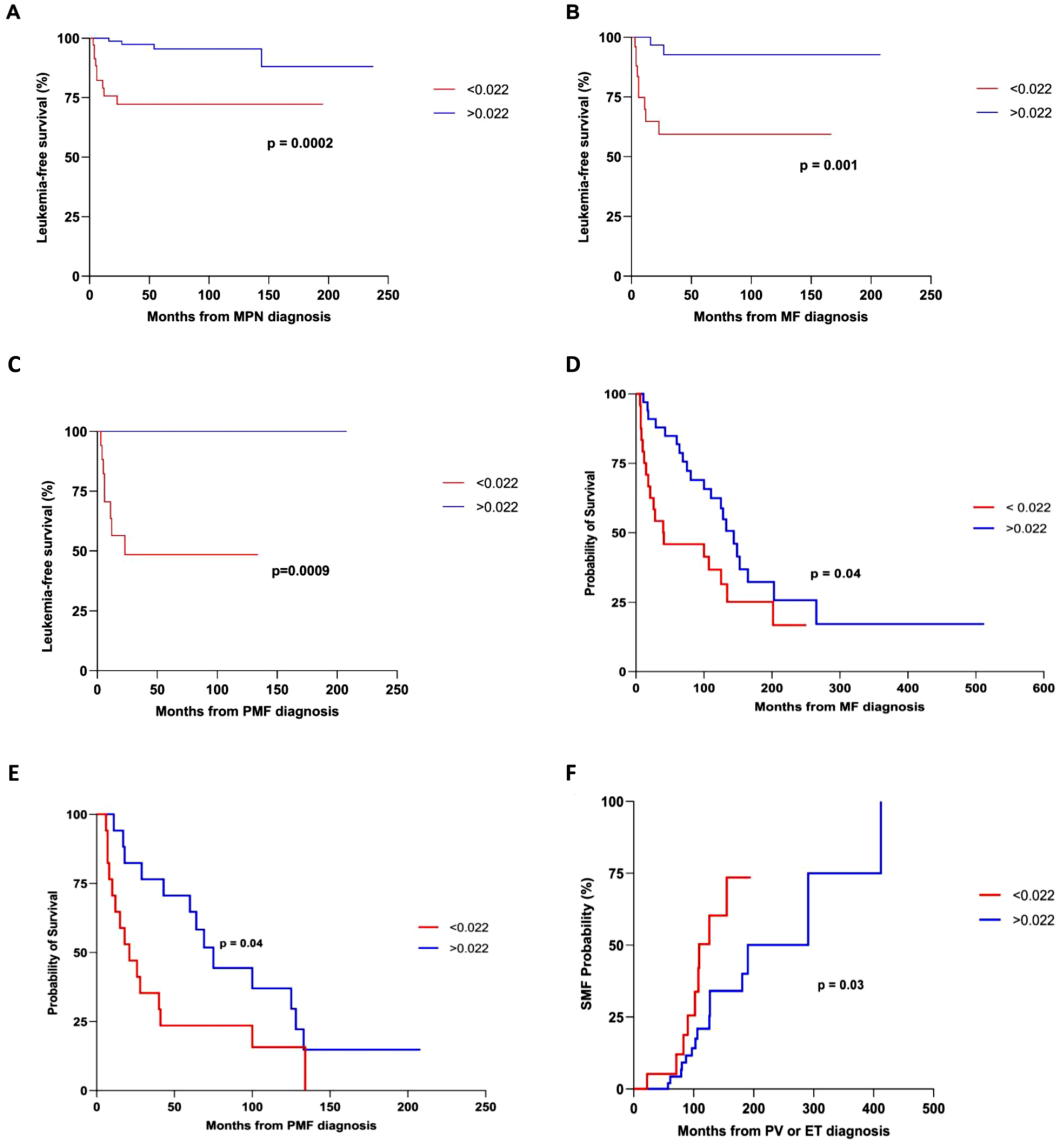
*IRF4* prognostic impact in Ph-MPNs. A LFS analysis in all MPN patients included in this study according to the *IRF4* expression value. **B** LFS analysis in MF patients according to the *IRF4* expression value. **C** LFS analysis in PMF patients according to the *IRF4* expression value. **D** OS analysis in MF patients according to the *IRF4* expression value. **E** OS analysis in PMF patients according to the *IRF4* expression value. **F** Probability analysis of evolution to post-PV and post-ET MF according to the *IRF4* expression value. The cut off of the *IRF4* expression (0.022) was calculated by ROC analysis

Among the 14 patients with LT, 9 (64.2%), 7 PMF and 2 SMF, were analyzed in next-generation sequencing [7] to detect the high molecular risk (HMR) mutations (Additional file 3: Table S2). In 5 PMF patients, HMR mutations were found; moreover, all 9 patients showed at least one additional genetic lesion besides the gene driver mutation (Additional file 4: Figure S1). Despite the data paucity, just under half of the patients with MF who had undergone LT (44.4%) did not have HRM mutations. In three PMF cases, the *IRF4* expression was evaluated during ruxolitinib treatment. All cases exhibited an increased *IRF4* expression compared to the value at diagnosis (p = 0.003); in the two patients with a longer duration of ruxolitinib treatment (59 mo. and 72 mo., respectively), there was a one-log increment of *IRF4* expression (Additional file 5: Figure S2).

Despite the main limits of our study (relatively small number of patients in each group, few NGS data, inability to determine the *IRF4* production source) we demonstrate an *IRF4* dysregulated expression in MPNs patients, particularly in PMF and in *JAK2* + and TN + cases, distinguishing those with a higher probability of SMF. Furthermore, the *IRF4* expression was associated with LT and a shorter LFS. Further studies are warranted to validate these data to confirm this biomarker as a new prognostic factor.

## Supplementary Information

**Additional file 1: Supplementary methods**. Detailed experimental procedures for droplet digital PCR, NGS and statistical analysis.

**Additional file 2: Table S1**. Main patients’ data. MPN patients biological and clinical characteristics.

**Additional file 3: Table S2**. NGS results. VCF file reporting the annotation of variants identified by NGS analysis.

**Additional file 4: Figure S1**. Oncoprinter visualization of NGS results. Variants identified for all cases analyzed (columns) are reported. The percentage value associated with each gene indicates its variants occurring in the cohort analyzed.

**Additional file 5: Figure S2**. Expression analysis of the IRF4 gene transcript at diagnosis and during ruxolitinib treatment in three myelofibrosis patients. The amount of IRF4 gene transcript was significantly increased during ruxolitinib therapy in all cases analyzed.

## Data Availability

Not applicable.

## Abbreviations

IRF4: Interferon regulatory factor 4
MDSCs: Myeloid-derived suppressor cells
MPNs: Myeloproliferative neoplasms
Ph-MPNs: Philadelphia negative myeloproliferative neoplasms
BM: Bone marrow
HC: Healthy controls
PMF: Primary myelofibrosis
ET: Essential thrombocythemia
TN: Triple negative
LT: Leukemic transformation
SMF: Secondary myelofibrosis
PV: Polycythemia vera
LFS: Leukemia-free survival
MF: Myelofibrosis
OS: Overall survival
HMR: High molecular risk
