# Supplementary material for: IRF4 expression is low in Philadelphia negative myeloproliferative neoplasms and is associated with a worse prognosis
Source: Exp Hematol Oncol. 2021 Dec 24;10:58. doi: 10.1186/s40164-021-00253-y (PMC8705160; doi:10.1186/s40164-021-00253-y)
Supplement: Supplementary file 1 — Additional file 1: Supplementary methods. Detailed experimental procedures for droplet digital PCR, NGS and statistical analysis. [file 40164_2021_253_MOESM1_ESM.docx]

**Supplementary methods**

***Patients***

One hundred nineteen newly diagnosed consecutive MPN-Ph- patients [20 polycythemia vera (PV), 40 essential thrombocythemia (ET), 35 primary myelofibrosis (PMF), and 23 secondary myelofibrosis (SMF)] were included in this study; their main clinical data are reported in Supplementary Table 1. The local ethics committee approved the study. Informed consent was obtained from all patients before study inclusion, in accordance with the Declaration of Helsinki. Patients' records/information were anonymized and de-identified before analysis. Consent for publication was obtained from patients before their enrolment in the present study.

***IRF4 expression evaluation***

At the disease onset, total bone marrow (BM) cells were isolated by whole blood lysis, and total RNA was extracted using the RNeasy Mini Kit (Qiagen, Hilden, Germany); the RNA concentration and purity were checked using the Qubit 2.0 fluorometer and Nanodrop UV-Vis spectrometer (Thermo Fisher Scientific, Waltham, MA). One µg of total RNA was reverse transcribed in complementary DNA (cDNA) using the QuantiTect reverse transcription kit (Qiagen).

According to the droplet digital PCR (ddPCR) Gene Expression EvaGreen Assays Protocol (BioRad, Hercules, CA), all assays were performed by adding to the QX200 ddPCR EvaGreen Supermix (BioRad, Hercules, CA), two specific primers (*IRF4*_F: GCCCAACAAACTGGAGAGAG and *IRF4*_R: CTGGAAACTCCTCTCCAAAGC , 50 ng of cDNA, in a final volume of 20 μl. Thermal-cycling conditions were 95°C for 5 min; 95°C for 30 s, 60°C for 1 min (40 cycles), 4°C for 5 min, 90°C for 5 min and 4°C hold. Furthermore, for each sample, quantification of glucuronidase b (*GUSB*) as a housekeeping gene [1] was conducted in a different well. Each reaction was replicated four times. Each experiment included a no-template control sample. ddPCR data were analyzed with QuantaSoft analysis software (version 1.7.4.0917). Results were considered valid when the number of accepted droplets per well was at least 10,000, and the housekeeping gene provided correct and reproducible amplification. The target quantification was calculated as the ratio between *IRF4* and *GUSB* number of copies (I/G). The patient's *IRF4* expression was compared with that of ten healthy controls. The *IRF4* median expression value of the healthy controls was also compared to that of a pool of 56 healthy control samples (Human Bone Marrow Total RNA-Clontech Laboratories, Inc., Mountainview, CA, USA); this latter comparison confirmed the overlapping *IRF4* range expression between the two control sets.

***Targeted next-generation sequencing (NGS)***

NGS analysis with an AmpliSeq customized panel (Thermo Fisher Scientific), encompassing 26 target genes that are frequently mutated in myeloid malignancies, was performed on genomic DNA extracted from BM samples, as previously described [2]. The targeted genes included *ASXL1*, *EZH2*, *IDH1*, *IDH2* and *SRSF2*. Quality control, reads alignment to the human genome (hg19) and variant calling (using the somatic workflow for single samples and the default parameters) were performed using Torrent Suite Software v5.12.2 (Thermo Fisher Scientific). Variants were annotated using Ion Reporter Software v5.18.0.2 (Thermo Fisher Scientific). Variants located in intronic regions (not in splice sites) or synonymous or present with >1% global minor allele frequency (MAF) in the healthy population, according to the Genome aggregation database (gnomAD) v2.1.1, were filtered out. Only variants with ≥2% variant allele frequency, and with a >500x depth of coverage were considered. Selected variants were investigated for a potential pathogenic role using the Catalogue of Somatic Mutations in Cancer (COSMIC v92) and dbSNP databases.

***Statistical analysis***

Continuous variables are presented as median (minimum-maximum). The Shapiro-Wilk test was performed to check the normal distribution of continuous variables. The differences in the distribution of continuous variables between categories were compared using the Mann–Whitney or Kruskal–Wallis test. Categorical variables were compared using Fisher’s exact test.

Overall survival (OS) was calculated from the diagnosis to the date of death or last follow-up. When calculating leukemia-free survival (LFS) and evolution to post-PV and post-ET myelofibrosis, the date of leukemic transformation and SMF diagnosis, respectively, were used instead of the date of death. OS and LFS analyses were performed with the Kaplan-Meier method, and the log-rank test was used to compare curves. The optimal cutoff value for discriminating leukemic transformation was defined using receiver operating characteristic (ROC) curve analysis. The predictive accuracy of the test was assessed by calculating the area under the curve (AUC). The Wilcoxon signed-rank test was used to compare, in two matched samples, the *IRF4* expression in the BM patient before and during ruxolitinib treatment. A p-value < 0.05 was considered statistically significant.

**References**

1. Cross NCP, White HE, Ernst T, Welden L, Dietz C, Saglio G, et al. Development and evaluation of a secondary reference panel for BCR-ABL1 quantification on the International Scale. Leukemia. 2016;30:1844–52.

2. Coccaro N, Zagaria A, Orsini P, Anelli L, Tota G, Casieri P, et al. RARA and RARG gene downregulation associated with EZH2 mutation in acute promyelocytic-like morphology leukemia. Hum Pathol. Elsevier Inc.; 2018;80:82–6.
