# Supplementary material for: IRF4 expression is low in Philadelphia negative myeloproliferative neoplasms and is associated with a worse prognosis
Source: Exp Hematol Oncol. 2021 Dec 24;10:58. doi: 10.1186/s40164-021-00253-y (PMC8705160; doi:10.1186/s40164-021-00253-y)
Supplement: Supplementary file 2 — Additional file 2: Table S1. Main patients’ data. MPN patients biological and clinical characteristics. [file 40164_2021_253_MOESM2_ESM.docx]

**Supplementary Table 1.** MPN patients biological and clinical characteristics. ASA: acetylsalicylic acid, HU: hydroxyurea, PT: phlebotomy, RUXO: ruxolitinib, IFN-α: recombinant interferon α.

|  | **Essential Thrombocythemia**  (n=40) | **Polycythemia Vera**  (n=20) | **Primary Myelofibrosis**  (n=35) | **Secondary Myelofibrosis**  (n=23) |
| --- | --- | --- | --- | --- |
| **Sex ratio, M/F** | 23/17 | 13/7 | 26/9 | 12/11 |
| **Median age (range), years** | 58 (18-81) | 58 (42-78) | 68 (20-85) | 67 (37-80) |
| **Risk Category*, n (%)**  **Treatment, n (%)** | Low=23 (57.5%)  High=17 (42.5%)  23 (57.5%) ASA, 17 (42.5%) ASA+HU | Low=10 (50%)  High=10 (50%)  9 (45%) ASA+PT, 11 (55%) ASA+HU | Low=2 (5.7%)  Int-1=14 (40%)  Int-2=13(37.1%)  High=6 (17.1%)  19 (54.3%) HU, 13 (37.1%) RUXO, 3 (8.6%) IFN-α | Low=4 (17.3%)  Int-1=8 (34.7%)  Int-2=7 (30.4%)  High=4 (17.3%)  8 (34.7%) HU, 15 (65.3%) RUXO |
| ***JAK2*, n (%)** | 23 (57.5%) | 20 (100%) | 19 (54.3%) | 12 (52.2%) |
| ***CALR*, n (%)** | 14 (35%) | 0 (0%) | 3 (8.6%) | 4 (17.4%) |
| ***MPL*, n (%)** | 0 (0%) | 0 (0%) | 2 (5.7%) | 2 (8.7%) |
| **Triple Negative, n (%)** | 3 (7.5%) | 0 (0%) | 11 (31.4%) | 5 (21.7%) |
| **Leukemic Transformation, n (%)** | 0 (0%) | 2 (10%) | 8 (22.8%) | 4 (17.4%) |

* For the risk category assessment, see text in the Results section.
