# Supplementary material for: IRF4 expression is low in Philadelphia negative myeloproliferative neoplasms and is associated with a worse prognosis
Source: Exp Hematol Oncol. 2021 Dec 24;10:58. doi: 10.1186/s40164-021-00253-y (PMC8705160; doi:10.1186/s40164-021-00253-y)
Supplement: Supplementary file 4 — Additional file 4: Figure S1. Oncoprinter visualization of NGS results. Variants identified for all cases analyzed (columns) are reported. The percentage value associated with each gene indicates its variants occurring in the cohort analyzed. [file 40164_2021_253_MOESM4_ESM.pdf]

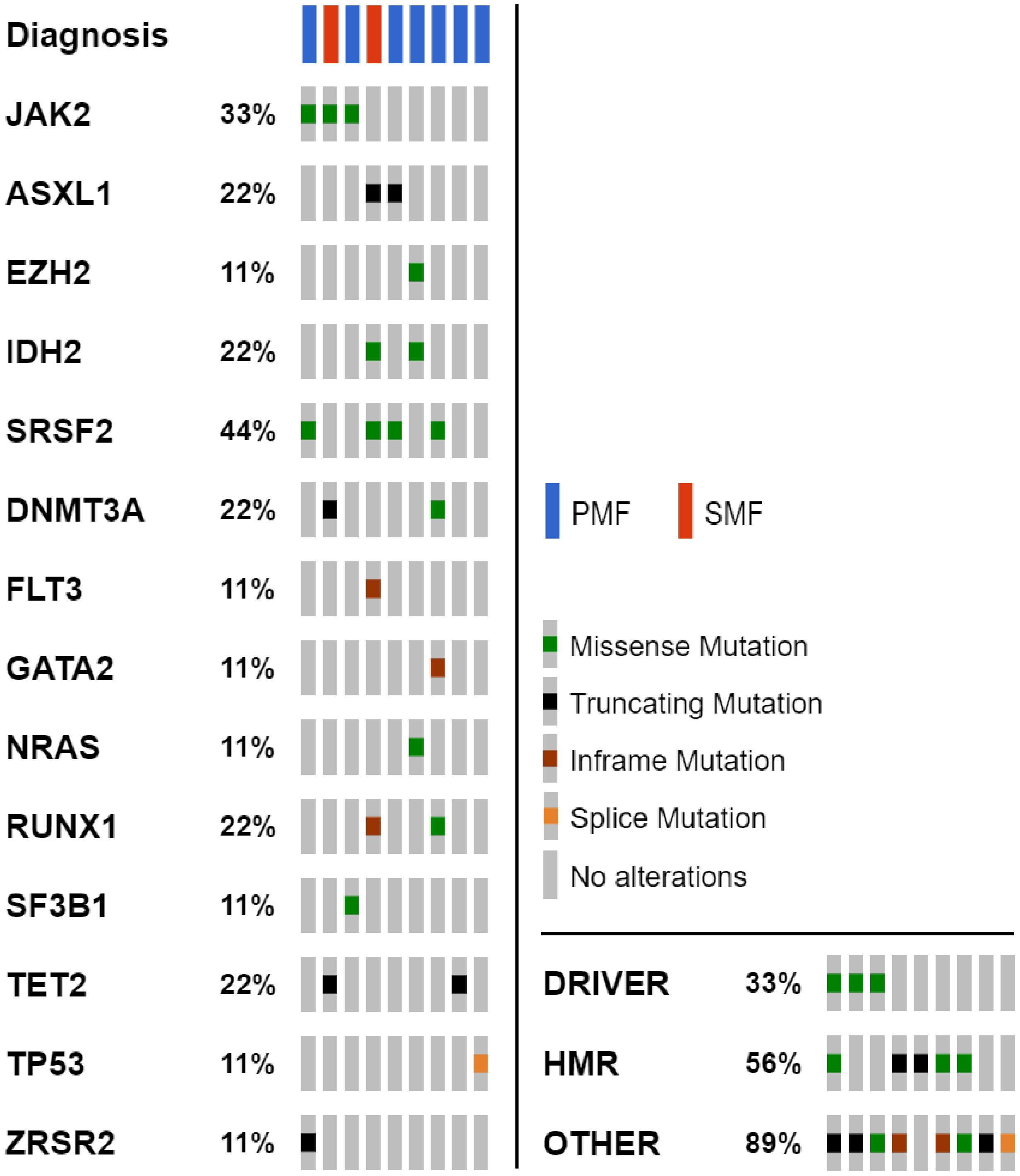
Diagnosis
JAK2 33%
ASXL1 22%
EZH2 11%
IDH2 22%
SRSF2 44%
DNMT3A 22%
FLT3 11%
GATA2 11%
NRAS 11%
RUNX1 22%
SF3B1 11%
TET2 22%
TP53 11%
ZRSR2 11%
PMF
SMF
Missense Mutation
Truncating Mutation
Inframe Mutation
Splice Mutation
No alterations
DRIVER 33%
HMR 56%
OTHER 89%
