# Supplementary figures and images for: IRF4 expression is low in Philadelphia negative myeloproliferative neoplasms and is associated with a worse prognosis
Source: Exp Hematol Oncol. 2021 Dec 24;10:58. doi: 10.1186/s40164-021-00253-y (PMC8705160; doi:10.1186/s40164-021-00253-y)

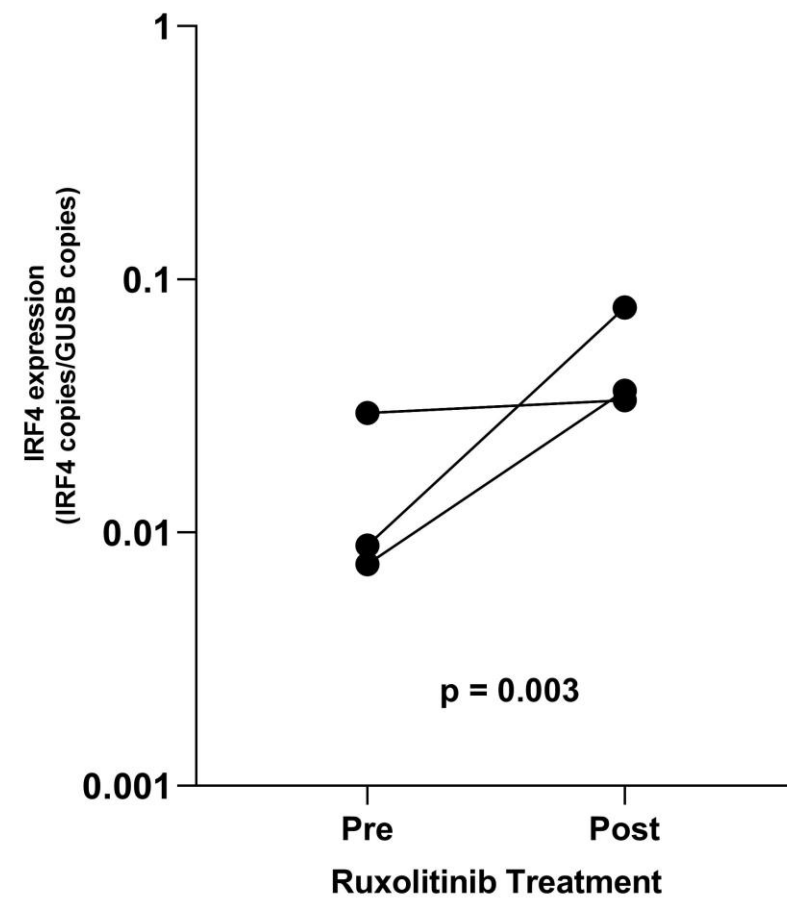

Supplement: Supplementary file 5 — Additional file 5: Figure S2. Expression analysis of the IRF4 gene transcript at diagnosis and during ruxolitinib treatment in three myelofibrosis patients. The amount of IRF4 gene transcript was significantly increased during ruxolitinib therapy in all cases analyzed. [file 40164_2021_253_MOESM5_ESM.pdf]
